# Optimal Placement of Accelerometers for the Detection of Everyday Activities

**DOI:** 10.3390/s130709183

**Published:** 2013-07-17

**Authors:** Ian Cleland, Basel Kikhia, Chris Nugent, Andrey Boytsov, Josef Hallberg, Kåre Synnes, Sally McClean, Dewar Finlay

**Affiliations:** 1 School of Computing and Mathematics, University of Ulster, Jordanstown, Co. Antrim, Northern Ireland BT37 0QB, UK; E-Mails: cd.nugent@ulster.ac.uk (C.N.); d.finlay@ulster.ac.uk (D.F.); 2 Department of Computer Science, Electrical and Space Engineering, Luleå University of Technology, Luleå 971 87, Sweden; E-Mails: basel.kikhia@ltu.se (B.K.); andrey.boytsov@ltu.se (A.B.); Josef.Hallberg@ltu.se (J.H.); Kare.Synnes@ltu.se (K.S.); 3 Computing and Information Engineering, University of Ulster, Coleraine, Co. Londonderry, Northern Ireland BT52 1SA, UK; E-Mail: si.mcclean@ulster.ac.uk

**Keywords:** activity recognition, accelerometery, wearable technology, classification models

## Abstract

This article describes an investigation to determine the optimal placement of accelerometers for the purpose of detecting a range of everyday activities. The paper investigates the effect of combining data from accelerometers placed at various bodily locations on the accuracy of activity detection. Eight healthy males participated within the study. Data were collected from six wireless tri-axial accelerometers placed at the chest, wrist, lower back, hip, thigh and foot. Activities included walking, running on a motorized treadmill, sitting, lying, standing and walking up and down stairs. The Support Vector Machine provided the most accurate detection of activities of all the machine learning algorithms investigated. Although data from all locations provided similar levels of accuracy, the hip was the best single location to record data for activity detection using a Support Vector Machine, providing small but significantly better accuracy than the other investigated locations. Increasing the number of sensing locations from one to two or more statistically increased the accuracy of classification. There was no significant difference in accuracy when using two or more sensors. It was noted, however, that the difference in activity detection using single or multiple accelerometers may be more pronounced when trying to detect finer grain activities. Future work shall therefore investigate the effects of accelerometer placement on a larger range of these activities.

## 1. Introduction

The ability to recognize various everyday activities provides new opportunities for context aware applications within a number of areas including healthcare and wearable computing. Activity recognition is based on the continuous monitoring of physical activity in free living environments for prolonged periods. In recent years, much work has been carried out on human activity recognition using wearable sensors. In particular, machine-learning techniques have been utilized to provide recognition of everyday activities, such as walking and lying [1,2], from accelerometer data. Their small size, light weight, low power consumption and low cost make accelerometers well suited to wearable applications [3]. With the increased use of accelerometers for activity detection, it is important to consider the technological challenges and limitations associated with their inaccuracies, placement issues and usability concerns.

**Figure 1.**
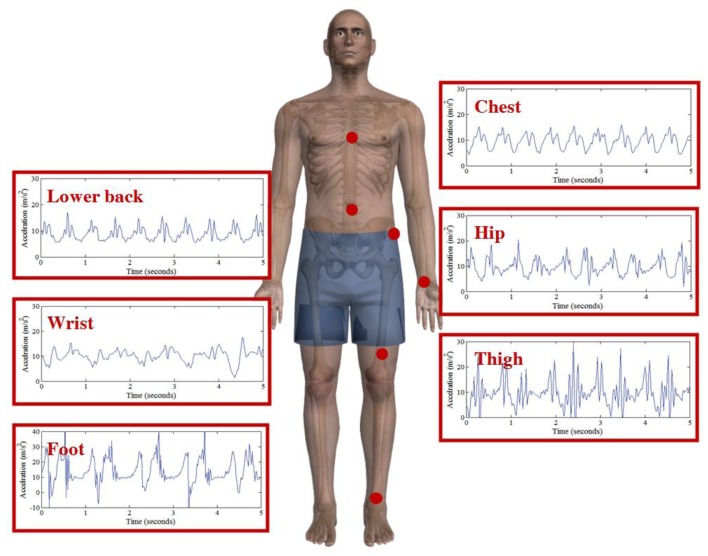
Five second recording of simultaneous vertical acceleration obtained from accelerometers placed on the chest, lower back, wrist, hip, thigh and foot. Acceleration data was sampled at 50 Hz using an accelerometer with a range of ±6 g.

The acceleration signal recorded from the body depends upon the location of the sensing device and the activity being performed [4]. Generally, acceleration signals are said to increase in magnitude from the head to the ankle. Vertical accelerations produced during level walking range from -2.9 m/s^2^ to 7.8 m/s^2^ at the lower back, to 16.7 m/s^2^ to 32.4 m/s^2^ at the tibia [4]. Figure 1 presents an illustration of typical accelerometer signals from chest, back, wrist, hip, thigh and foot whilst walking. Although it is agreed that accelerometer placement has an effect on the measurement of bodily acceleration, there is still some debate over the ideal location of the sensor for particular applications [5].

This paper reports an investigation into the optimal placement of accelerometers for the detection of everyday activities. Activities investigated within this study include walking, jogging on a motorized treadmill, sitting, lying, standing and walking up and down stairs. Data is collected from 6 locations on the body, namely the chest, left hip, left wrist, left thigh, left foot and lower back. Machine-learning techniques are utilized to identify which location is best to place accelerometers for the purpose of activity detection. In addition, the authors compare the activity detection accuracy when combining the acceleration data from different locations. This work aims to answer the following research questions:

1. What is the best machine-learning model for classifying the investigated activities from the acceleration data?
2. What is the optimal location of a single tri-axial accelerometer for detecting the selected range of everyday activities?
3. How does combining tri-axial accelerometers located at different locations affect the accuracy of activity detection?

The remainder of this article is organized as follows: Section 2 presents a review of related works which have investigated accelerometer placement for detecting everyday activities. In Section 3 the methods utilized within this work are discussed. The methods section includes data capture, processing and analysis. The results from experimentation, with respect to each of the research questions, are then discussed in Section 4. Finally conclusions are drawn in Section 5.

## 2. Related Work

Accelerometers are widely integrated into wearable systems in order to identify various activities. Activity detection aims to identify activities based on data collected from ubiquitous sensors as they occur. The ability to provide accurate information on a user's activity and context lends itself to numerous application areas including, activity monitoring/promotion, context aware information and content delivery. Previous studies investigating activity detection have reported accuracy levels of 85% to 95% for recognition rates during ambulation, posture and activities of daily living (ADL). A summary of notable works is presented in Table 1. From the Table, it is worth noting that there is a large variety of placement locations utilized within these projects. Furthermore, the majority of these studies have incorporated multiple accelerometers attached at different locations on the body. Whilst this provides sufficient contextual information, placing accelerometers in multiple locations can become cumbersome for the wearer, which can impact on wearer compliance. Increasing the number of sensors also increases the complexity of the classification problem. For these reasons, a number of studies have investigated the use of a single accelerometer. However, doing so generally decreases the number of activities that can be recognized accurately [6]. In light of this, one of the major considerations in using accelerometers for activity detection is to identify which location, or combination of locations, on the body provides the most relevant information to perform the detection. Bao and Intille [7] used features derived from both the time and frequency domain to classify 20 different activities. Accelerometers were placed at the upper arm, lower arm, hip, thigh and ankle. Data generated from the accelerometers was used to train a number of classifiers including the C4.5 Decision Tree, Decision Tables, Naive Bayes and Nearest Neighbor classifier. The Decision Tree classifier yielded the best performance achieving 86% accuracy. Further analysis showed a reduction in accuracy of just 3% when data from only the thigh and the wrist was considered. Although this work is seen as seminal in the area of activity recognition, it did not report on how the location of the accelerometer affected the accuracy of the classifier for each activity. Furthermore, all possible combinations of sensors were not investigated.

A study by Olguin and Pentland [8], compared the activity recognition accuracy of four configurations of accelerometers from three placements. The mean and variance of the three axes were used as inputs to a Hidden Markov model (HMM). Validation was carried out using a 9-fold cross validation. The classifier achieved an accuracy of 65% using only one accelerometer placed at the chest. By combining data from accelerometers placed on the wrist and hip, this accuracy increased to 87%. Additionally, using data from all three locations improved the classification accuracy to 92%. In agreement with the work by Bao and Intille, the authors suggested that reasonably accurate activity recognition (∼80%) could be achieved using a system consisting of two accelerometers and highlighted that this could be made up of an electronic badge and a mobile phone. As with similar studies only a small subset of possible combinations of sensors where investigated. There is therefore a need to investigate all possible combinations of sensors.

In addition to investigation of optimum placement of accelerometers for ambulatory activities and activities of daily living, a small number of studies have invetsigated the effects of placement on the detection of specific activities such as falls. Gjoreski *et al.* [9] studied the best location to place accelerometers for fall detection, based on the classification of postures. Four accelerometers were placed at the chest, waist, ankle and thigh. Statistical features were calculated for each axis of the accelerometer in addition to the magnitude. Results indicated that one accelerometer (chest or waist) by itself was not enough to sufficiently classify the activities (75%). There was, however, a significant improvement in classification accuracy achieved by combining the accelerometer at the chest or waist with one placed on the ankle (91%). In agreement with other studies, the authors found using sensors placed on both the upper and lower body improved the classification of the activity. One limitation to this study is that they did not investigate ambulatory activities (walking, running, stair climbing). Furthermore, the authors [9] did not report on the classification accuracy for all possible combinations of sensors.

Other studies have looked to investigate which features, obtained from multiple accelerometers provide the most discriminative power. Preece *et al.* [10] addressed the area of dynamic activity recognition and the specific challenge of extracting relevant features from the accelerometer signal. Recognized activities included walking, going up and down the stairs, running, hopping on the left or right leg and jumping. Accelerometers were placed on the waist, ankle and thigh of the participants. Preece *et al.* analyzed various time-domain and frequency-domain features of accelerometer signals, in addition to multiple sets of features based on using a wavelet transform. In order to compare different feature sets, the authors used the k Nearest Neighbor (kNN) classifier with an Euclidean distance metric. The highest activity recognition accuracy for a single sensor (97%) was achieved using data from an accelerometer placed at the ankle. Preece *et al.* noted surprisingly high levels of accuracy, compared to other works when using frequency-domain features derived from a single accelerometer. Furthermore, frequency domain features were shown to outperform both time-domain and Wavelet based feature sets. The creation of these frequency based features, derived by a fast Fourier transform is, however, extremely resource intensive.

More recently, Atallah *et al.* [2] investigated the optimal placement of accelerometers for classifying groups of activities. Accelerometers were placed at seven locations including the chest, hip, upper arm, wrist, thigh, ankle and ear, this is the greatest number of locations to be investigated. They assessed how kNN and Bayesian classifiers performed on features obtained from each location. From this they made recommendations for choosing which location was best for different activity levels, e.g., very low, low, medium, high and transitional. Sensors on the wrist provided reasonably good rates of precision and recall for similar activities, e.g., preparing food and eating and drinking. An accelerometer placed at the waist performed best for low level activities, where the differences in body acceleration were more distinctive, e.g., walking and reading. This most recent study, however, did not assess any effect that combining accelerometers may have on the performance of the classifier.

Many previous studies have assessed the effects of placement using conventional locations such as the hip and lower back. More recently, however, some studies have highlighted the need to consider convenient placement of sensors in order to improve compliance [11]. Indeed, compliance is a large source of data loss within free living studies. Nevertheless, best practices for the use of activity monitors still suggest that monitors should be worn in a comfortable, unobtrusive location and firmly attached to the body [12]. Other factors which are associated with user compliance include sensor size, weight, number and attachment method [12]. Bergmann *et al.* [11] investigated agreement in the accelerometer feature, median frequency, for sensors fixed at the lower back and placed within the front pocket. Twelve subjects were asked to complete four tasks including, standing, walking and climbing stairs. Only accelerations from the vertical axis showed moderate agreement. The authors suggested that the generalizability between traditional and convenient placements may therefore be limited. This study, however, only considers a small number of sensor placements and does not assess the effect that the difference in placement had on classification accuracy.

This article describes an investigation into the optimal placement of accelerometers for the purpose of activity detection. Furthermore, the authors investigate the effect of combining data from accelerometers at multiple locations on the accuracy of activity detection. Although previous works have investigated the accuracy of activity detection from each of these locations, few have used data from more than five locations [2,7]. Of these studies, none have considered all possible combinations of sensor locations. This work shall therefore clarify which combination of sensors provides the greatest accuracy of detection of everyday activities.

## 3. Methods

The following section describes the protocol for the collection of accelerometer data for the investigated activities. Following this, details of the features extracted from the raw data and the models used to carry out the activity recognition are described.

### 3.1. Data Collection

**Figure 2.**
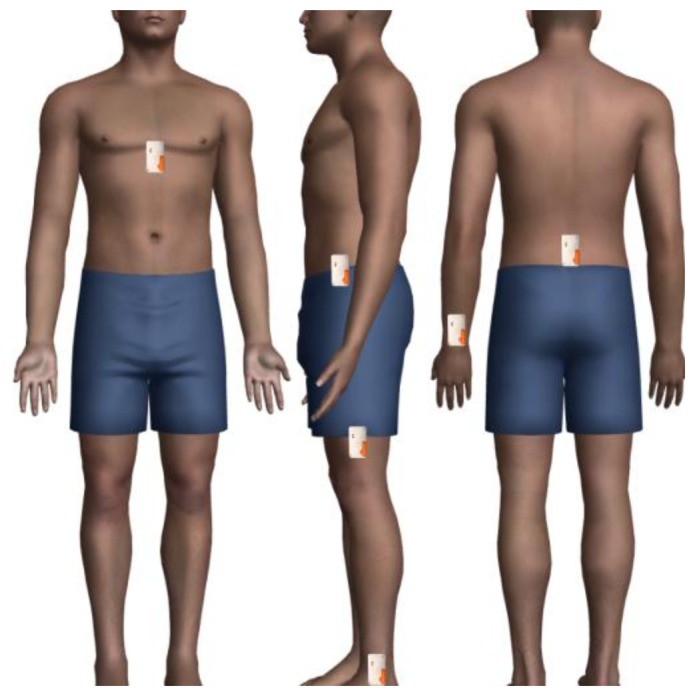
Selected placement locations for the accelerometers. These include the chest, lower back, hip, thigh, wrist and foot. Accelerometers were fixed on top of clothing using elasticated strapping and holsters.

Eight male subjects were recruited to participate in the study. Subjects were members of staff and students of the University of Ulster. Subjects ranged in age from 24 to 33 (mean 26.25, sd ± 2.86). All subjects provided written informed consent prior to participating in the study. Subjects also completed a physical activity readiness questionnaire (PAR-Q) to assess their suitability to take part in the study. The study was approved by the Faculty of Computing and Engineering Research Governance Filter Committee at the University of Ulster. Subjects wore six tri-axial accelerometers at different locations on the body as shown in Figure 2. These locations were selected as they are typical sites from which to collect data for the purpose of activity recognition as shown by Table 1. Furthermore, these sites have been shown to provide accuracy rates of ∼80% for whole-body ambulatory activates similar to those within this study. Accelerometers were fixed to the body using elasticized strapping and holsters. This is a common method of attachment in activity recognition studies [3].

Acceleration data was collected using six Shimmer wireless sensor platforms (Shimmer 2R, Realtime Technologies, Dublin, Ireland). These tri-axial accelerometers had a range of ±6 g and sampled data at 51.2 Hz. This sampling frequency is sufficient to capture most everyday activities (∼20 Hz) [3]. Bodily acceleration amplitude can range up to ±1 2 g. Nonetheless, the literature suggests that promising results can be obtained using ±2 g acceleration data during activity recognition [7]. Furthermore, although acceleration at body extremities can exhibit a 12 g range in acceleration, the majority of points near the torso and hip experience only a 6 g range in acceleration [3].

Data were transmitted via Bluetooth to a notebook computer where it was saved for analysis offline. In order to achieve synchronization, data were recorded using Shimmer Sync software (Shimmer sync Version 1.0). This software synchronizes time stamp data from each of the 6 accelerometers. Prior to beginning the study, devices were calibrated using standard calibration techniques as described in [21].

Seven activities were studied. These consisted of whole body activities and postures including walking, jogging on a motorized treadmill, sitting, lying, standing and walking up and down stairs. All activities were maintained for a duration of two minutes with the exception of climbing stairs. The stair climbing activities were carried out on 10 flights of stairs (∼80 steps). The climbing stairs task was repeated, after a one minute pause, in order to capture sufficient data for analysis. For treadmill based activities, users walked and jogged at a self-selected comfortable speed. The maximum jogging speed was capped at 10 km/h as speeds above this are considered as running [22]. Data were manually labeled offline by a human observer. A summary of average times to complete tasks along with walking and running speeds is presented in Table 2.

### 3.2. Feature Extraction

The raw acceleration data were labeled based on the performed activity. There were approximately 50,000 samples for each activity with a total number of 370,000 samples. Features were extracted from raw acceleration data using a window size of 512 samples with 256 samples overlapping between consecutive windows. Feature extraction on windows with a 50% overlap has demonstrated reasonable results in previous works [7]. This window size is capable of capturing complete cycles in repetitive action activities such as walking, jogging and stair walking, whilst allowing for fast computation of features. Eleven features were extracted from each window, giving a total of 26 attributes. A description of each feature is presented in Table 3. These features have been used within previous works and have achieved acceptable levels of accuracy (∼80%) [7,17].

Feature 1–8 are standard statistical metrics. Feature 9 (Energy) is the sum of the squared discrete FFT component magnitudes of the signal [23]. The sum is divided by the window length for the purposes of normalization (1). This feature has been shown to result in accurate detection of certain postures and activities [24]. For instance, the energy of a subject's acceleration can discriminate low intensity activities such as lying from moderate intensity activities such as walking and high intensity activities such as jogging [25]. If ×1, ×2, … are the FFT components of the window then the energy can be represented as presented in Equation (1):

(1) $${\text{Energy}}_{\text{x}}=\frac{\sum_{\text{i}=1}^{\left|\text{w}\right|}{\left|{\text{x}}_{\text{i}}\right|}^{2}}{\left|\text{w}\right|}$$

where *x_i_* are the FFT components of the window for the *x* axis and *w* is the length of the window. Feature 11 (Correlation) has been shown to improve the detection of activities involving movements of multiple body parts [26]. It is helpful for differentiating among activities that involve translation in just one dimension [17]. For instance, with correlation between axes it is possible to differentiate walking and jogging from taking the stairs up and down. Correlation is calculated between each pair of axes as the ratio of the covariance and the product of the standard deviations [17], Equation (2):

(2) $$\text{corr}\left(\text{x},\text{y}\right)=\frac{\mathrm{cov}\left(\text{x},\;\text{y}\right)}{\sigma_{\text{x}}\sigma_{\text{y}}}$$

where cov(x,y) is the ratio of covariance between the x and y axis of acceleration and σ*_x_*σ*_y_* is the product of the standard deviations. The features are used as input for WEKA data mining software (University of Waikato, Version 3.6.7) to build the classifiers. WEKA is a machine learning software environment which offers a collection of visualization tools and algorithms for data analysis and predictive modeling [27].

### 3.3. Classification Models

Many different classification models have been applied to the problem of activity detection. This is highlighted by the studies included in Table 1. There is no universally accepted method of detecting a particular range of activities and all techniques have associated benefits and limitations. Common methods include data driven approaches such as Decision Trees (DT), k-nearest Neighbor, Neural Networks (NN), Naive Bayes (NB) and Support Vector Machine (SVM) [28]. An overview of techniques and their associated benefits and limitations is provided by Preece *et al.* [23]. In order to identify which machine learning algorithm provided the most accurate activity detection, the C4.5 DT (J48), NB, NN (Multilayer Perceptron) and SVM were applied to data. The parameters of each classification method were configured by identifying the set of parameters that correspond to maximum average accuracy over a 10-fold cross validation. A java application was developed to perform a grid search for optimal parameters. To further identify which machine learning algorithm achieved the best accuracy, a 10 fold cross validation with 10 iterations was performed using the WEKA Experimenter (University of Waikato, Version 3.6.7). A paired t-test was subsequently performed on the results to identify if the percentage of correctly classified instances was significantly different using the SVM when compared to the J48, NB or NN. SPSS (IBM, Version 20) was used for all statistical tests. The SVM was used as the baseline scheme, with the other three algorithms being compared to it. A value of less than *p* = 0.05 was considered statistically significant.

## 4. Results and Discussion

### 4.1. Accuracy of Classification Algorithms

In order to identify which machine learning algorithm provided the most accurate activity detection, the Decision tree (J48), Naïve Bayes (NB), Neural Network (NN) (Multilayer Perceptron) and Support Vector Machine (SVM) where applied to the data. The parameters of each classification method were configured by identifying the set of parameters that correspond to maximum average accuracy over a 10-fold cross validation. A java application was developed to perform a grid search for optimal parameters. For the J48 algorithm, the application compared the performance of different confidence values (with a step of 1%) combined with the use of different values of minimum number of instances (in increments of one). For the J48 the best results were achieved using a confidence value of 5% and the minimum number of instances equal to 2. For the NN, the application analyzed the optimal number of layers and number of neurons per layer in the multilayer perceptron. A model with 70 neurons in a single hidden layer provided best average recognition accuracy. For the SVM, the application compared the performance of different kernels and different complexity values. The best performance was achieved with universal Pearson VII function based kernel [25] and complexity value of 100. For the NB method supervised discretization was used [26], which significantly increased the performance of the approach. Table 4 presents the percentage of the correctly classified instances for each location using the adopted machine learning methods.

Results in Table 4 demonstrate that the SVM provided significantly better accuracy than the DT, NB and NN when using data from all locations, with the exception of data from the foot, where the DT and NB provided statistically better results. There was no significant difference in percentage classified instances between the SVM and NN when using data from the hip or thigh. The average percentage classified instances for all locations was used as a final metric to compare overall performance of the approaches. SVM provided a small, however, significantly greater accuracy of 96.67% (*p* ≤ 0.001). SVM is a popular classification technique, which has previously been shown to provide accurate activity detection [29]. SVMs can be implemented in real time and work reliably on noisy data sets [23]. For these reasons the SVM was selected as the method of choice for subsequent testing.

### 4.2. Optimal Location of Single Accelerometer

Table 4 also provides details of which location provided data that achieved the most accurate activity detection. A one way ANOVA, with Tukey post hoc testing, was performed to assess statistical significance. From Table 4 it can be observed that the highest accuracy was achieved when using data from the Hip (97.81%, *p* ≤ 0.001). The lowest accuracy was achieved when using data from the Foot (95.63, *p* ≤ 0.001) and Wrist (95.88, *p* = 0.002). In this case, the Hip is therefore the best single location to place a tri-axial accelerometer for detecting the studied range of everyday activities.

The F-measure was used as a performance index to evaluate the SVMs ability to classify each of the activities. The F-measure combines *precision* and *recall* as presented in Equation (3):

(3) $$\text{F}-\text{measure}=2\times \frac{\text{precision}\times \text{recall}}{\text{precision}+\text{recall}}$$

*Precision* is the fraction of retrieved instances that are relevant, while *Recall* is the fraction of relevant instances that are retrieved. A higher F-measure value indicates improved detection of the investigated activity. Table 5 presents the balanced F-measure calculated for each activity at each location when using the SVM. Again a 10 fold cross validation was applied. A weighted average value was added to the Table to represent the average of the F-measure values for each location.

Results show activity detection using data from the hip provided the highest F-measure with an average of 0.978, while data from the foot provided the lowest F-measure average of 0.955. It is hypothesized that the hip provided the best data, as the activities studied do not consist of arm or upper body movements and as data from the hip best represents total body movement. In addition to the weighted average value of F-measure, it is possible to identify the accuracy of detecting each activity separately. For example, sitting and standing activities were detected worst when using data from the left foot, the values are 0.924 and 0.929 respectively, which indicates some confusion of detecting those activities when attaching the accelerometer to the left foot. This is most likely due to little or no difference in the accelerometer signal recorded at the foot during these activities. Lying and jogging activities were the most accurately detected activities. Data from the wrist and lower back provided similarly low average F-measures. For the data from the wrist this lower accuracy may be due, in part, to arm movements unassociated with the measured activity. For example, some users sat with their arms folded while others gestured and talked with their hands. When using accelerometers placed at the lower back it was noticed that the accelerometer tended to twist and rotate on the elasticated strapping. This in turn may have caused irregularities within the data, which subsequently impacts negatively on the classification of activities when using data from this location.

### 4.3. Optimal Location of Multiple Accelerometers

All possible combinations of two, three, four, five and six locations were generated. Considering the total number of combinations for all six locations studied produces 64 possible permutations in total (2^6^ = 64). Removing from this, cases involving single or no sensors resulted in 57 (64–1–6 = 57) possible combinations. For reporting purposes these combinations were divided as follows: 15 possible combinations for two locations, 20 possible combinations for three locations, 15 possible combinations for four locations, six possible combinations for five locations and one possible combination for all six locations. After computing the features, files were used as input for the WEKA Experimenter. The SVM classifier was then applied. A 10 fold cross-validation with 10 iterations was again applied. Tables 6, 7, 8, 9 and 10 present the results, in terms of accuracy, for combinations of two to six locations, respectively. A one way ANOVA was performed in order to assess whether or not the location of the accelerometer had a statistically significant impact on the percentage of correctly classified instances.

**Figure 3.**
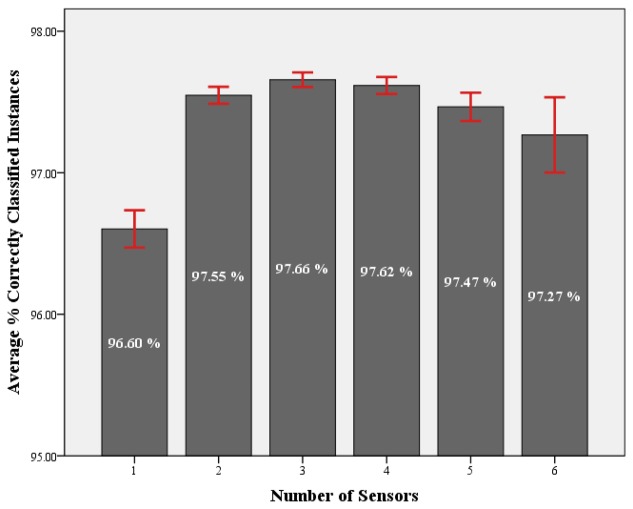
Graph presents the average percentage of correctly classified instances for each number of sensor combinations. The average for each number of sensors is labeled. Error bars represent 95% confidence intervals.

Results from Tables 6, 7, 8 and 9 demonstrated that there was no significant difference in accuracy, depending on the location of the sensors, when using combinations of two (*p* = 0.074), three (*p* = 0.409), four (*p* = 0.727) or five (*p* = 0.788) sensors. In order to investigate the effect of combining accelerometers from multiple locations on the accuracy of activity detection, the average accuracy when using each number of sensors was compared. Figure 3 presents the average accuracy for each number of sensors. Once again a one way ANOVA, with Tukey post hoc testing, was performed in order to assess the significance of the results.

There was a small, however, significant increase in accuracy achieved by increasing the number of sensing locations from one to two or more sensors (*p* ≤ 0.001). No significant improvement in performance was noticed when combining data from more than two locations (*p* = 0.25). There was a small, however, significant decrease in classification accuracy when using data from five or more locations compared to that achieved using data from three (*p* = 0.012) or four (*p* = 0.07) locations. When investigating placement of wearable technology it is also important to consider issues of wearability and user comfort. Key considerations include where the device can be placed to allow it to function correctly while not impinging on a user's activity. Carrying multiple devices also impacts or usability as the wearer must charge and carry multiple devices ensuring they are worn correctly. This can reduce compliance acceptance of the technology. With these results in mind and considering the issues of wearability and usability, which would indicate the use of fewer sensors to be preferable, it can be concluded that the optimal number of sensors for detecting the range of activities investigated is two sensors. These sensors can, however, be placed at any of the investigated locations and will most likely depend upon the types of activities which are being investigated as illustrated by Table 5.

There are a number of limitations which have come to light as a result of carrying out this study. Within the experimental protocol only whole body activities such as walking, standing and sitting are considered. Other finer grain activities, such as sitting reading or sitting eating, may rely on identifying further movements in order to distinguish between activites and are not represented by whole body movements. For this reason the variety of activities studied should be expanded to include these lower level actives. Furthermore, data used within this study was captured within a laboratory under controlled conditions. It may therefore not be representative of carrying out such activities in a free living environment. Future studies should therefore seek to carry out further experiments using data captured under free living conditions by utilizing mobile and pervasive computing technologies.

Lastly, this study considered activity classification using only data from accelerometers. Recent studies have shown that introducing data from a variety of sensor types can improve the classification accuracy of everyday activities [30]. Future work should therefore consider the effects of placement on the classification accuracy from sensors such as gyroscopes and magnetometers.

## 5. Conclusions

This paper presented an investigation into the accuracy of activity detection from accelerometer data recorded simultaneously from six bodily locations. Results have shown that the SVM provided the most accurate classification results of the investigated machine learning algorithms, when using data from a single location. Data from the hip was shown to be the best single location for providing data to detect the range of activities. Although, the differences in classification accuracy between locations were found to be significant, they are in fact reasonably close and therefore the practical implications of this are marginable. This study further investigated the effect of combining multiple accelerometers from various locations. In doing so, it was shown that reasonable activity detection can be achieved using only two accelerometers and that increasing the number of sensors had no significant impact on the accuracy of the classifier. This was in line with previous works, which have shown that an accelerometer placed on the upper and one on the lower part of the body can successfully detect a range of everyday activities [7]. Previous studies did not, however, investigate all possible combinations of sensors.

Furthermore, results within the current study show that the difference in accuracy of activity detection for systems with using one and multiple accelerometers is less than previously reported. These results may, however, be due to the type of activity that was investigated in the current study. Activities within this study focused on high level activities, such as walking lying and sitting, which are grossly different from each other in terms of acceleration signals. If finer grain activities were to be studied which have more subtle differences in acceleration, such as for example sitting and sitting working at a computer, then these results may have shown greater differences between using a single or multiple accelerometers. Further work should therefore focus on identifying which combination of accelerometer positions provides the best accuracy for these finer grain activities. Additionally, the accuracy of such classifiers should be assessed under more free living conditions.

## Figures and Tables

**Table 1.** Summary of notable works involving activity detection using accelerometry. The table includes the type of activities which were investigated (total number of activities is given in brackets), the number of subjects (n), the features used and detection accuracy achieved.

**Reference**	**Activities (Number Studied)**	**n**	**Accelerometer Placements**	**Features**	**Accuracy**
Bao and Intille [7]	Walking, sitting, running, cycling, vacuuming, folding laundry (20)	20	Upper arm, lower arm, hip, thigh, foot	Mean, entropy, energy	Decision tree (84%), kNN (83%), Naive Bayes (52%)
Karantonis [13]	Sitting, Standing, walking, lying in various positions and falls (12)	6	Waist	Signal magnitude area, tilt angle, signal magnitude vector	Decision tree (91%)
Pirttikangas [14]	Typing, watching TV, drinking, walking up and down stairs (17)	13	Both wrists, thigh and necklace	Mean, standard deviation	Neural network (93%) kNN (90%)
Mathie [15]	Fall, walking, transitional, sit, stand and lie (6)	26	Waist	Signal magnitude area, mean acceleration	Decision tree (87%)
Parkka [16]	Lying sitting, walking, rowing, cycling (8)	11	Chest and wrist	Mean, variance, median, skewness, kurtosis, peak frequency, signal power	Decision tree (86%) Hierarchical (82%) Neural network (82%)
Olguin and Pentland [8]	Sitting, Running, walking, standing, lying and crawling (7)	3	Chest, hip, wrist	Mean and variance	HMM (65%–92%)
Ravi [17]	Standing, walking, running, stairs up, stairs down, vacuuming (8)	2	Waist	Mean, Standard deviation, energy, correlation Mean, Standard deviation, peak-to-peak	Naive bayes (64%) SVM (63%) Decision tree (57%) kNN (50%)
Bonomi [18]	Lying, sitting, standing, working on a computer, walking, running, cycling (7)	20	Lower back	distance, cross-correlation, spectral power, dominant frequency	Decision tree (93%)
Yeoh [19]	Sitting, lying, standing and walking speed (4)	5	Waist and thigh	Accelerometer inclination	Heuristic model (100%)
Yang [1]	Standing, sitting, walking, running, vacuuming, scrubbing brushing teeth (8)	7	Wrist	Mean, correlation, energy, interquartile range, RMS	Neural network (95%) kNN (87%)
Lyons [20]	Sitting, standing, lying, moving (4)	1	Trunk and Thigh	Mean, standard deviation and inclination	Thresh holding (93%)
Gjoreski [9]	Lying, sitting, standing, all fours, transitional (7)	11	Chest, Waist, Ankle, Thigh	Orientation, Mean, Root Mean Square, Standard Deviation and Movement detection	Random Forest (75%–99%)
Atallah [2]	Lying, walking, running, cycling, sitting, transitional (15)	11	Chest, upper arm, wrist, hip thigh, ankle, ear	Variance, RMS, mean, energy, entropy, skewness, kurtosis, covariance	kNN (na), Bayesian (na)

**Table 2.** Summary of the time taken to complete walking and stair walking tasks and mean speed for walking and running on a treadmill. Figures presented are average and ± standard deviation.

**Activity**	**Mean Time to Complete (s)**	**±Standard Deviation**
Stairs up	49.38	(±6.74)
Stairs down	45.31	(±4.88)

	Average Speed (km/h)	± Standard Deviation
	
Walking speed	4.63	(±0.34)
Running speed	8.44	(±0.98)

**Table 3.** Description of features extracted from each window of raw acceleration data. 11 features were extracted from each window, giving a total of 26 attributes.

**No.**	**Feature Description**
1	Mean value for each axis (x, y, and z)
2	Average Mean over 3 axes
3	Standard Deviation value for each axis (x, y, and z)
4	Average Standard Deviation over 3 axes
5	Skewness value for each axis (x, y, and z)
6	Average Skewness over 3 axes
7	Kurtosis value for each axis (x, y, and z)
8	Average Kurtosis over 3 axes
9	Energy value for each axis (x, y, and z)
10	Average Energy over 3 axes
11	Correlations: x_y, x_z, x_total, y_z, y_total, z_total

**Table 4.** Percentage of correctly classified instances for each location using each of the four machine learning algorithms. Results show the average percentage correctly classified instances for the 10 fold 10 iteration test. P-values are presented in brackets. (*) denotes significantly less than percentage correctly classified instances, (+) denotes significantly more than percentage correctly classified instances and (-) denotes no significant difference in percentage correctly classified instances. The average percentage accuracy for all locations is also presented.

**Location**	**SVM**	**J48**		**NB**		**NN**	
		
		**% Correct**	**(P-value)**	**% Correct**	**(P-value)**	**% Correct**	**(P-value)**
Chest	96.91	94.22	(<0.001) *	92.5	(<0.001) *	95.34	(<0.001) *
Foot	95.63	96.48	(<0.001) +	97.42	(<0.001) +	93.94	(<0.001) *
Left Hip	97.81	94.11	(<0.001) *	95.92	(<0.001) *	97.75	−0.57 -
Lower back	96.59	92.8	(<0.001) *	94.91	(<0.001) *	95.77	(<0.001) *
Left Thigh	96.81	94.6	(<0.001) *	96.35	−0.012 *	96.85	−0.751 -
Left Wrist	95.88	92.87	(<0.001) *	91.52	(<0.001) *	94.81	(<0.001) *
Average	96.67	94.18	(<0.001) *	94.77	(<0.001) *	95.74	(<0.001) *

**Table 5.** Balanced F-measure for each location, detailed by class, when using the Neural Network. A weighted average value was added to the Table to represent the average of the F-measure values for each location.

**Activity**	**Chest**	**Lower Back**	**Left Foot**	**Left Hip**	**Left Thigh**	**Left Wrist**
Lying	1	1	0.997	1	0.972	0.967
Running	1	1	1	1	1	1
Sitting	0.966	0.992	0.924	1	0.972	0.966
Stairs down	0.94	0.92	0.915	0.935	0.925	0.926
Stairs up	0.928	0.906	0.92	0.929	0.929	0.902
Standing	0.969	0.993	0.929	1	1	1
Walking	0.981	0.973	1	0.99	1	0.961
Weighted	0.9	0.968	0.955	0.978	0.971	0.965
Avg.	69					

**Table 6.** Percentage correctly classified instances for 2 location combinations.

**Chest**	**Lower Back**	**Left Foot**	**Left Hip**	**Left Thigh**	**Left Wrist**	**Accuracy**
X	X					97.30%
X		X				97.71%
X			X			97.65%
X				X		97.84%
X					X	97.79%
	X	X				97.31%
	X		X			97.38%
		X	X			97.48%
			X	X		97.71%
			X		X	97.48%
	X			X		97.74%
		X		X		97.61%
	X				X	97.47%
		X			X	97.61%
				X	X	97.30%

**Table 7.** Percentage correctly classified instances for 3 location combinations.

**Chest**	**Lower Back**	**Left Foot**	**Left Hip**	**Left Thigh**	**Left Wrist**	**Accuracy**
X	X	X				97.68%
X	X		X			97.57%
X		X	X			97.57%
X			X	X		97.73%
X			X		X	97.55%
X	X			X		97.91%
X		X		X		97.85%
X	X				X	97.55%
X		X			X	97.77%
X				X	X	97.85%
	X	X	X			97.73%
	X		X	X		97.70%
		X	X	X		97.64%
	X		X		X	97.46%
		X	X		X	97.57%
			X	X	X	97.54%
	X	X		X		97.74%
	X	X			X	97.48%
	X			X	X	97.65%
		X		X	X	97.62%

**Table 8.** Percentage correctly classified instances for 4 location combinations.

**Chest**	**Lower Back**	**Left Foot**	**Left Hip**	**Left Thigh**	**Left Wrist**	**Accuracy**
X	X	X	X			97.66%
X	X		X	X		97.78%
X		X	X	X		97.59%
X	X		X		X	97.43%
X		X	X		X	97.66%
X			X	X	X	97.62%
X	X	X		X		97.73%
X	X	X			X	97.53%
X	X			X	X	97.68%
X		X		X	X	97.66%
	X	X	X	X		97.75%
	X	X	X		X	97.52%
	X		X	X	X	97.52%
		X	X	X	X	97.58%
	X	X		X	X	97.54%

**Table 9.** Percentage Correctly Classified Instances for 5 location combinations.

**Chest**	**Lower Back**	**Left Foot**	**Left Hip**	**Left Thigh**	**Left Wrist**	**Accuracy**
X	X	X	X	X		97.63%
X	X	X	X		X	97.42%
X	X		X	X	X	97.40%
X		X	X	X	X	97.43%
X	X	X		X	X	97.46%
	X	X	X	X	X	97.46%

**Table 10.** Percentage correctly classified instances for 6 location combinations.

**Chest**	**Lower Back**	**Left Foot**	**Left Hip**	**Left Thigh**	**Left Wrist**	**Accuracy**
X	X	X	X	X	X	97.26%
